# X-ray absorption spectroscopy of FeH^+^ to aid its identification in astrochemical environments†

**DOI:** 10.1039/d4cc04702h

**Published:** 2024-12-17

**Authors:** Shan Jin, Max Flach, Alexander Ebenbichler, Ethan M. Cunningham, Vicente Zamudio-Bayer, Konstantin Hirsch, Christian van der Linde, Norbert Przybilla, Milan Ončák, J. Tobias Lau, Martin K. Beyer

**Affiliations:** a Universität Innsbruck, Institut für Ionenphysik und Angewandte Physik, Technikerstraße 25/3 6020 Innsbruck Austria martin.beyer@uibk.ac.at; b Abteilung für Hochempfindliche Röntgenspektroskopie, Helmholtz-Zentrum Berlin für Materialien und Energie Albert-Einstein-Str. 15 12489 Berlin Germany; c Physikalisches Institut, Albert-Ludwigs-Universität Freiburg Hermann-Herder-Str. 3 79104 Freiburg Germany; d Universität Innsbruck, Institut für Astro- und Teilchenphysik, Technikerstr. 25/8 6020 Innsbruck Austria norbert.przybilla@uibk.ac.at

## Abstract

We present the first absorption spectrum of the unperturbed diatomic molecular ion FeH^+^ in any wavelength range. The cryogenic X-ray absorption spectrum at the L_2_ and L_3_ edge is consistent with an iron 3d occupation of 6.24e. Comparison with the interstellar absorption spectrum of Cygnus X-1 indicates that FeH^+^ cannot be ruled out as a component of the absorbing medium.

## Abstract 2

High-resolution X-ray absorption spectroscopy is performed on the diatomic molecular ion FeH^+^ and compared with the absorption of the interstellar medium in the iron L_2,3_ edge observed in the Cygnus X-1 spectrum.

## Graphical abstract

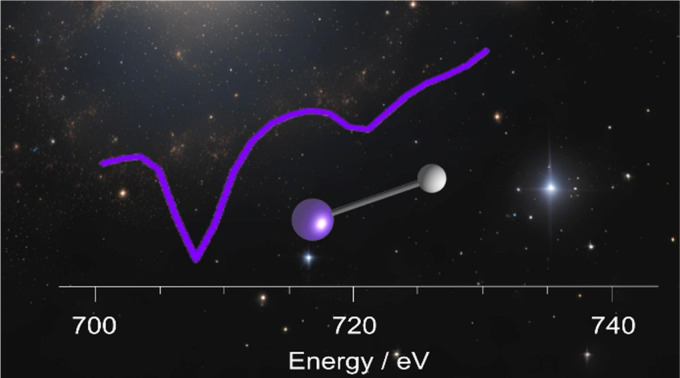

With hydrogen as the most abundant element and iron the second most abundant metal in the Milky Way,^1^ the diatomic molecular ion FeH^+^, among other iron containing molecular ions,^2^ has long been suspected to be present in stellar atmospheres or in the interstellar medium (ISM).^3,4^ In the absence of laboratory data, however, it has not been identified so far in any astronomical environment. The only laboratory spectrum to date is the infrared photodissociation spectrum of Ar_2_FeH^+^ in the Fe–H stretch region.^5^ Due to the shift induced by argon tagging, however, it is not suitable for comparison with data from astronomical observations.

Interstellar X-ray absorption spectroscopy in comparison with laboratory data has been used to assign the chemical form of iron in the ISM, and its fractionation into gas phase and dust particles.^6–8^ With no laboratory XAS data available, it has so far not been possible to consider iron containing molecular species like FeH^+^ in such studies. Here we report a spectrum of the iron L_2,3_ edges of FeH^+^ in the gas phase. It is the first laboratory spectrum of FeH^+^ in any spectral region that is suitable for comparison with data from astronomical observations. We also briefly compare the results with recent spectra of diatomic iron-halide cations^9^ to discuss the implications of the spectrum for the electronic structure of FeH^+^, in particular the d-shell occupation, which so far has been studied only computationally.^4,10,11^

X-ray absorption spectroscopy (XAS) was performed at the IonTrap station of the UE52-PGM beamline at the BESSY II electron storage ring operated by the Helmholtz-Zentrum Berlin für Materialien und Energie.^12^ An electrospray ionization source with an ion funnel interface^13^ is used to produce FeH^+^ from dimethyl ferrocene in aqueous solution (see ESI† for details). This efficient way of making FeH^+^ was motivated by earlier work, which used electron ionization of dimethyl ferrocene.^14,15^ Mass selected FeH^+^ was trapped in a linear Paul trap and collisionally cooled to *T* ≈ 10 K by using cryogenic helium buffer gas.^16^ X-ray absorption spectra with photon energy calibration with an uncertainty of less than 0.2 eV were recorded in partial ion yield mode,^16,17^ see ESI† for details. Supporting quantum chemical calculations were performed in Gaussian.^18^ Local 3d occupation was obtained by charge transfer multiplet (CTM) simulations using CTM4XAS,^19^ and compared with quantum chemical calculations. As usual, an empirical shift was applied to match the peak position of CTM4XAS calculations with experiment.

Fig. 1 summarizes the XAS spectra of FeH^+^, with the spectra of Fe^+^ shown for comparison.^9^ The L_3_ edge of FeH^+^ exhibits less structure than the atomic ion, and the peak maximum is shifted to slightly smaller energies. However, the median L_3_ energies at 708.1 eV are identical within the error bars, with a slightly higher uncertainty for FeH^+^ due to the smaller signal intensity for the molecular ion. The L_2_ edge also stays at the same energy, with an overall similar peak shape. CTM simulations reproduce the overall spectral shape very well, with respect to peak broadening and substructure of the L_2_ and L_3_ edges. It should be noted that the CTM4XAS code and the underlying routines were developed for crystals in the solid state. Therefore the representation of the *C*_∞v_ point group of FeH^+^ by a crystal field in *C*_4_ symmetry is not perfect.

**Fig. 1:**
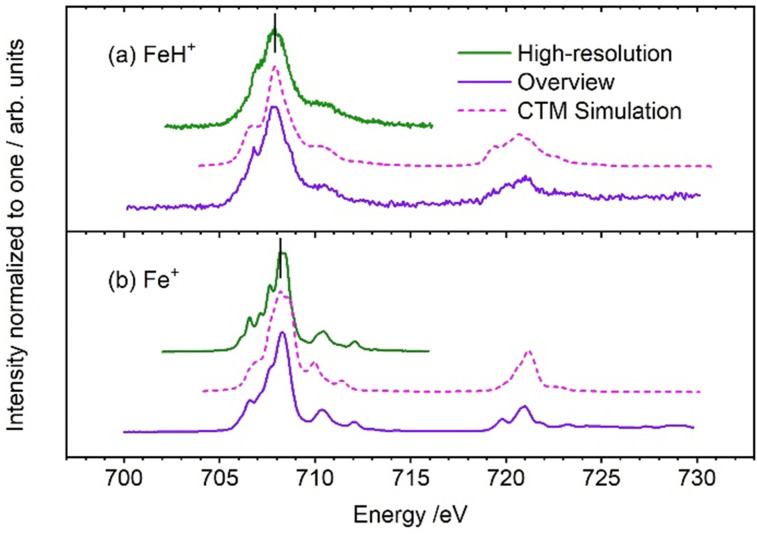
Experimental iron L_3,2_ edge XAS spectra of (a) FeH^+^ (this work) and (b) Fe^+^ (reproduced from Flach *et al.*, ref. 9), compared with CTM calculations (dashed lines). The black vertical lines in the figure are the median values of the experimental L_3_ intensity distribution.

**Fig. 2:**
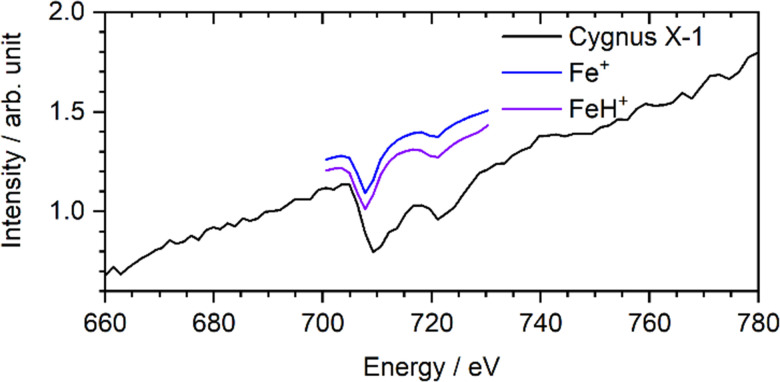
Comparison of interstellar XAS spectrum with laboratory spectrum of Fe^+^ and FeH^+^ at the iron L_2,3_ edges, after adding an empirical baseline and further processing (see text for details). The interstellar spectrum is taken from the XMM-Newton Science Archive.^20^ The XMM-Newton RGS observations 0745250601 and 0745250501 were coadded.

In our earlier work on iron halides,^9^ we observed a strong correlation between the median position of the L_3_ edge and the Mulliken electronegativity of the halogen^9,21–24^ as well as the 3d population of the iron center in FeX^+^, X = F, Cl, Br, I. Although both FeH^+^ and FeX^+^ contain a formal single bond and have ^5^Δ ground state, their electronic structure is completely different: one bonding σ molecular orbital is doubly occupied in FeH^+^, while doubly and singly occupied π, π* and σ* molecular orbitals contribute to the bonding in FeX^+^. To test whether the electronegativity correlation still holds despite these differences in electronic structure, we performed quantum chemical calculations. Tables S1 and S2 (ESI†) summarize Mulliken electronegativities and iron 3d and 4s populations obtained by a natural bond orbital analysis (NBO) with density functional theory (DFT). Obviously, the electronegativity correlation breaks down, while the 3d population prevails as the relevant property. CTM simulations yield an iron 3d population of 6.24, Table S3 (ESI†), in agreement with the values obtained from NBO analysis, Table S2 (ESI†).

In Fig. 2, we compare the Fe^+^ and FeH^+^ spectrum with observational data provided by the XMM-Newton Science Archive of the European Space Agency (ESA),^20^ using spectra obtained *via* the reflecting grating spectrometer (RGS). Our laboratory spectra were converted to absorption spectra, convolved with a Gaussian kernel with the instrumental resolution of the RGS, binned to the RGS binning, and an arbitrary scaling and a simplified baseline were applied. The XMM-Newton data captures the total extinction, so both absorption and scattering, but scattering is negligible.^6^ This processing of the laboratory data more or less removes the subtle differences between the Fe^+^ and FeH^+^ laboratory spectra.

Since our model spectra agree in the onset and the lower-energy, but not in the higher-energy, part of the iron L_3_ and L_2_ edges of the interstellar spectrum, it is obvious that Fe^+^, FeH^+^, or any iron(ii) species, alone cannot be the carrier of the L_2,3_ edge absorption in the ISM along this sightline, as shown before,^6,7^ but that oxidation states of iron higher than +2 have to be present because the median excitation energy shifts to higher values with increasing oxidation state.^25^ However, the interstellar X-ray absorption spectrum results from all iron compounds which are present in the interstellar medium. Since there is no feature in the FeH^+^ spectrum that would not be covered by the absorptions in the Cygnus X-1 data, the presence of the FeH^+^ molecular ion in the ISM cannot be ruled out. The similarity of the processed Fe^+^ and FeH^+^ spectrum in Fig. 2 underlines that FeH^+^ may contribute to the gas-phase component of interstellar X-ray absorption at the iron L_2,3_ edges.^26^ However, the main carriers of the absorption obviously are other iron containing molecules or molecular ions, clusters or small particles.

With the X-ray absorption spectrum of FeH^+^ we present the first laboratory data for this elusive species that can be directly compared with astronomical observations. Besides its astrochemical relevance, the spectrum carries information on the population of iron 3d orbitals in this molecular ion. Parameters for CTM simulations have been found that yield 3d occupations similar to quantum chemical NBO analysis. There is a clear need for X-ray reference data of iron species to identify further candidates for the carriers of interstellar X-ray spectra.

Shan Jin: data curation (supporting), investigation (equal), visualization (equal), writing – review and editing (supporting); Max Flach: conceptualization (equal), data curation (lead), formal analysis (lead), investigation (equal), writing – original draft (supporting), writing – review and editing (supporting); Alexander Ebenbichler: data curation (supporting), formal analysis (supporting), investigation (supporting), visualization (equal), writing – review and editing (supporting); Ethan M. Cunningham: funding acquisition (supporting), investigation (equal), project administration (supporting), resources (supporting), supervision (supporting); Vicente Zamudio-Bayer: investigation (supporting), writing – review and editing (supporting); Konstantin Hirsch: investigation (supporting), writing – review and editing (supporting); Christian van der Linde: methodology (supporting), writing – review and editing (supporting); Norbert Przybilla: data curation (supporting), funding acquisition (supporting), investigation (supporting), supervision (equal); Milan Ončák: data curation (supporting), formal analysis (supporting), investigation (equal), methodology (equal); J. Tobias Lau: conceptualization (equal), funding acquisition (equal), methodology (equal), writing – review and editing (supporting); Martin K. Beyer: conceptualization (equal), formal analysis (supporting), funding acquisition (equal), supervision (equal), writing – original draft (lead).

Beamtime for this project was granted by HZB at beamline UE52-PGM Ion Trap of the BESSY II synchrotron radiation facility. JTL and MF acknowledge support by Deutsche Forschungsgemeinschaft (DFG) within RTG 2717. The computational results presented have been achieved using the HPC infrastructure LEO of the University of Innsbruck. This research was funded in part by the Austrian Science Fund (FWF), Grant DOIs 10.55776/W1259 (JS, AE, NP, MKB) and 10.55776/M3027 (EMC). For open access purposes, the author has applied a CC BY public copyright license to any author accepted manuscript version arising from this submission. The authors thank Professor Frank de Groot for access to the CTM4XAS code and helpful advice on simulating iron L_2,3_ edge XAS spectra.

## Data availability

The data supporting this article have been included as part of the ESI.†

## Conflicts of interest

There are no conflicts to declare.

## Supplementary Material

CC-061-D4CC04702H-s001

CC-061-D4CC04702H-s002
