# Supplementary material for: X-ray absorption spectroscopy of FeH+ to aid its identification in astrochemical environments
Source: Chem Commun (Camb). 2024 Dec 17;61(10):2048–50. doi: 10.1039/d4cc04702h (PMC11694246; doi:10.1039/d4cc04702h)
Supplement: CC-061-D4CC04702H-s002 [file CC-061-D4CC04702H-s002.pdf]

Supplementary Information (SI) for ChemComm.
This journal is © The Royal Society of Chemistry 2024

Electronic Supplementary Information

X-ray absorption spectroscopy of FeH^+ to aid its identification in astrochemical environments

Shan Jin,[a] Max Flach,[b,c] Alexander Ebenbichler,[d] Ethan M. Cunningham,[a] Vicente Zamudio-Bayer,[b] Konstantin Hirsch,[b] Christian van der Linde,[a] Norbert Przybilla,[*,d] Milan Ončák,[a] J. Tobias Lau[b,c] and Martin K. Beyer[*,a]

[a] *Universität Innsbruck, Institut für Ionenphysik und Angewandte Physik, Technikerstraße 25/3, 6020 Innsbruck, Austria*
[b] *Abteilung für Hochempfindliche Röntgenspektroskopie, Helmholtz-Zentrum Berlin für Materialien und Energie, Albert-Einstein-Str. 15, 12489 Berlin, Germany.*
[c] *Physikalisches Institut, Albert-Ludwigs-Universität Freiburg, Hermann-Herder-Str. 3, 79104 Freiburg, Germany*
[d] *Universität Innsbruck, Institut für Astro- und Teilchenphysik, Technikerstr. 25/8, 6020 Innsbruck, Austria*

E-mail: Norbert.Przybilla@uibk.ac.at; martin.beyer@uibk.ac.at

Table S1. Electronegativities of several elements. All electronegativities χ were determined from ionization energies (IE) and electron affinities (EA) according to Mulliken.[1] Electronegativities of F, Cl, Br, and I are taken from Flach et al.[2]

Element	IE/eV[3]	EA/eV	χ/eV
H	13.59844	0.75497[4]	7.18
F	17.42282	3.448[5]	10.44
Cl	12.967633	3.613[5]	8.29
Br	11.81381	3.363[5]	7.59
I	10.451260	3.063[5]	6.76

Table S2. Partial charge on the iron atom *q*(Fe) calculated using the CHELPG method and electron population in Fe atomic orbitals *p*(AO) as obtained through the natural bond orbital analysis in the structure optimized at the respective level. Various DFT functionals were employed along with the def2QZVP basis set. The following non-default atomic radii were used in the CHELPG calculations: 1.94 Å (Fe), 1.85 Å (Br), 1.98 Å (I).

method	property	FeH^+	FeF^+	$FeCl^+$	$FeBr^+$	FeI^+
B3LYP+D3	*q*(Fe)	1.22	1.42	1.18	1.07	0.94
	p(4s)	0.45	0.10	0.19	0.25	0.33
	p(3d)	6.29	6.28	6.39	6.42	6.46
	p(4p)	0.01	0.04	0.07	0.08	0.09
	p(4d)	0.00	0.00	0.01	0.01	0.01
CAM-B3LYP	*q*(Fe)	1.24	1.45	1.21	1.11	0.96
	p(4s)	0.43	0.09	0.18	0.23	0.48
	p(3d)	6.28	6.26	6.35	6.37	6.25
	p(4p)	0.02	0.04	0.08	0.09	0.12
	p(4d)	0.00	0.00	0.01	0.01	0.02
M06	*q*(Fe)	1.25	1.45	1.20	1.08	0.86
	p(4s)	0.46	0.08	0.18	0.23	0.54
	p(3d)	6.23	6.27	6.37	6.41	6.34
	p(4p)	0.02	0.03	0.07	0.08	0.10
	p(4d)	0.01	0.01	0.01	0.02	0.03
*ω*B97XD	*q*(Fe)	1.27	1.46	1.23	1.12	0.96
	p(4s)	0.40	0.08	0.17	0.21	0.46
	p(3d)	6.28	6.27	6.35	6.38	6.26
	p(4p)	0.02	0.04	0.08	0.09	0.13
	p(4d)	0.00	0.01	0.01	0.01	0.02

Experimental set up and methods

The experiment was performed at the IonTrap endstation of the UE52-PGM beamline at the BESSY II synchrotron radiation facility.[6] The details were presented in the previous research.[2] We describe it briefly here. We produce the ions of interest using electrospray ionization with an ion funnel interface.[7,8]

1,1'-Dimethylferrocen(95%) is a commercial product from Sigma-Aldrich. The sample is dissolved in methanol and water (9:1) and diluted for the electrospray ionization (ESI) source. The formation of FeH^+ from 1,1'-Dimethylferrocen by electron ionization (EI) has been reported before.[9,10] The ionization mechanism in ESI and EI is certainly different, but since both ESI and EI yield the molecular cation, we saw a chance that the FeH^+ fragment can be obtained under harsh ESI conditions. This turned out to be the case.

FeH^+ cations are mass selected using a quadrupole mass filter and are subsequently guided via radio frequency quadrupole and hexapole ion guides into a linear Paul trap for accumulation.[6] The trap is cryogenically cooling ions to a temperature of ~10 K by collisions with helium buffer gas.[11] After excitation of the precursor with X-rays, photofragments are generated and subsequently extracted into a time-of-flight mass spectrometer. We recorded the dominant partial ion yield channel Fe^{2+}. Hence the partial ion yield can be considered to be proportional to the X-ray absorption cross-section of the precursor.[12,13]

The photon energy is scanned in steps of 30 meV, at a bandwidth of 80 meV during high-resolution measurements, and in steps of 80 meV, with a bandwidth of 200 meV for overview measurements. The iron 2p core hole lifetime broadening is approximately 400 meV,[14,15] higher than the irradiation bandwidth, hence the multiplet structure at the L_3 edge can be resolved in both measurements.

The absolute energy calibration of the beamline monochromator was performed at the beginning of the beam time, using the neon 1s excitation at 867.29 eV together with the three diffraction orders of the grating. The accuracy of this calibration is limited to 0.2 eV due to reproducibility and long term stability of the monochromator angles. To guarantee comparability with our previous data sets of FeX^+ (X=F,Cl,Br,I),[2] reference measurements of the iron L_3-edge of $FeCl^+$ were taken.

CTM Simulations

We performed charge transfer multiplet simulations using the CTM4XAS code[16] and extracted local 3d occupations from the best fit to the experimental spectrum. Due to the large parameter space, we imposed some constraints, motivated by the physics of the two ions. In general, $F_{pd} = G_{pd}$ is a good approximation, and F_{dd} is significantly smaller than F_{pd}, G_{pd}. All three parameters are usually 1.0 or lower. Spin-orbit coupling in iron is still small in the valence shell, values around 0.1-0.3 worked well in our case. Following the findings of Richter et al. that the 4s electron does not have a noticeable effect,[17] neither crystal field splitting nor charge transfer was invoked for Fe^+. For FeH^+, the C_4 point group is closest to $C_{\infty v}$. Δ was judiciously chosen as the difference of the lowest states of the $3d^6 4s^1$ and $3d^7$ configurations of Fe^+. Among the CT hopping parameters, only the T(a1) is non-zero, because charge transfer is only possible along the bonding σ molecular orbital of FeH^+, involving $3d_{z2}$ and 4s orbitals of Fe and 1s of H. Otherwise, the parameters were manipulated until a qualitatively satisfactory match with experiment was obtained. The parameters for the best fit to the Fe^+ and FeH^+ spectra are given in Table S3.

Table S3. Parameters of charge transfer multiplet simulations shown in Figure 1, together with the resulting 3d occupation, *p*(3d). Here, F_{dd}, F_{pd} and G_{pd} describe the slater integral reduction; SOC refers to the valence spin-orbit coupling reduction; crystal field splitting $10D_q$ in C_4 symmetry for FeH^+; charge transfer parameters are Δ as the energy difference between the center of the $3d^6$ and $3d^7$ configurations, the Hubbard parameter U_{dd}, the core hole potential U_{pd} and hopping parameter $T(a_1)$; empirical shift of simulated spectrum to higher energies for positive sign.

Parameter	FeH^+	Fe^+
F_{dd} in %	0.8	0.85
F_{pd} in %	0.9	0.95
G_{pd} in %	0.9	0.95
SOC in %	0.2	0.2
$10D_q$ in eV	0.4	-
Δ in eV	0.23	-
U_{dd} in eV	0.2	-
U_{pd} in eV	1.2	-
T(a1) in eV	1.3	-
p(3d) in e	6.24	6.00
Empirical shift in eV	-0.046	0.145

Data treatment for comparison with XMM-Newton Data

We used the XMM-Newton observations 0745250601 and 0745250501 of the X-ray binary Cygnus X-1. The two observations were taken close in time and show similar spectra. To enhance the signal-to-noise ratio, we calculated the average flux of those observations, a procedure known as coaddition in astronomy. In this step, the Cygnus X-1 spectra were normalized to arbitrary intensity units. The resulting coadded spectrum is our astronomical comparison data. Resolution of XMM-Newton is about 2.5 eV, while the accuracy of the energy scale is about 0.2 eV in the energy range shown in Figure 2.

To make the laboratory FeH^+ data comparable, we had to apply a few changes. The ion yield intensity at the photon energy E is proportional to the absorption cross section σ(E) of FeH^+. We calculated an absorption spectrum f(E) from σ(E) using the formula:

$$f(E) = (a + E*b) * \exp(-n * \sigma(E))$$

Here a and b are baseline parameters and n is a scaling factor for the interstellar FeH^+ column density.

To match the resolutions of the XMM-Newton and laboratory data, we convolved f(E) with a Gaussian kernel to simulate the low resolution of XMM-Newton. The width of the kernel is ΔE = 2.5 eV. To match the energy binning, we rebinned the convolved spectrum to the binning of the XMM-Newton data.

The resulting spectrum is compared to the Cygnus X-1 observations. It should be noted that no shift was applied on the energy scale, since the accuracy of both laboratory XAS and XMM-Newton energies is 0.2 eV, an order of magnitude smaller than the width of the binning.

References

1 R. S. Mulliken, *J. Chem. Phys.*, 1934, **2**, 782.

2 M. Flach, K. Hirsch, M. Timm, O. S. Ablyasova, M. da Silva Santos, M. Kubin, C. Bülow, T. Gitzinger, B. von Issendorff, J. T. Lau and V. Zamudio-Bayer, *Phys. Chem. Chem. Phys.*, 2022, **24**, 19890.

3 A. Kramida, Y. Ralchenko and Reader, J., NIST ASD Team, *NIST Atomic Spectra Database (ver. 5.8), [Online].,* available at: http://physics.nist.gov/asd, accessed 30 August 2021.

4 R. C. Shiell, X. Hu, Q. J. Hu and J. W. Hepburn, *Faraday Discuss.*, 2000, **115**, 331-343.

5 R. S. Berry and C. W. Reimann, *J. Chem. Phys.*, 1963, **38**, 1540.

6 K. Hirsch, J. T. Lau, P. Klar, A. Langenberg, J. Probst, J. Rittmann, M. Vogel, V. Zamudio-Bayer, T. Möller and B. von Issendorff, *J. Phys. B: At. Mol. Opt. Phys.*, 2009, **42**, 154029.

7 D. Egorov, B. Sadia, R. Hoekstra, A. Lawicki, K. Hirsch, V. Zamudio-Bayer, T. Lau, B. von Issendorff and T. Schlathölter, *J. Phys.: Conf. Ser.*, 2015, **635**, 112083.

8 S. Bari, D. Egorov, T. L. C. Jansen, R. Boll, R. Hoekstra, S. Techert, V. Zamudio-Bayer, C. Bülow, R. Lindblad, G. Leistner, A. Ławicki, K. Hirsch, P. S. Miedema, B. von Issendorff, J. T. Lau and T. Schlathölter, *Chem. Eur. J.*, 2018, **24**, 7631.

9 E. I. Mysov, I. R. Lyatifov, R. B. Materikova and N. S. Kochetkova, *J. Organomet. Chem.*, 1979, **169**, 301.

10 L. F. Halle, F. S. Klein and J. L. Beauchamp, *J. Am. Chem. Soc.*, 1984, **106**, 2543.

11 V. Zamudio-Bayer, R. Lindblad, C. Bülow, G. Leistner, A. Terasaki, B. von Issendorff and J. T. Lau, *J. Chem. Phys.*, 2016, **145**, 194302.

12 M. Ahmed, K. Asmis, I. Avdonin, M. K. Beyer, E. Bieske, S. Bougueroua, C.-W. Chou, S. Daly, O. Dopfer, L. Ellis-Gibbings, V. Gabelica, M.-P. Gaigeot, M. Gatchell, B. Gerber, C. Johnson, M. Johnson, K. Jordan, A. Krylov, M. Mayer, L. McCaslin, A. B. McCoy, D. Neumark, M. Ončák, J. Oomens, A. Rijs, T. Rizzo, J. Roithová, P. Sarre, S. Schlemmer, J. Simons, M. Stockett, A. Trevitt, J. Verlet, X.-B. Wang, R. Wester, S. Willitsch and S. Xantheas, *Faraday Discuss.*, 2019, **217**, 138.

13 J. T. Lau, J. Rittmann, V. Zamudio-Bayer, M. Vogel, K. Hirsch, P. Klar, F. Lofink, T. Möller and B. von Issendorff, *Phys. Rev. Lett.*, 2008, **101**, 153401.

14 K. Hirsch, V. Zamudio-Bayer, F. Ameseder, A. Langenberg, J. Rittmann, M. Vogel, T. Möller, B. von Issendorff and J. T. Lau, *Phys. Rev. A*, 2012, **85**.

15 P. Gambardella, S. S. Dhesi, S. Gardonio, C. Grazioli, P. Ohresser and C. Carbone, *Phys. Rev. Lett.*, 2002, **88**, 47202.

16 E. Stavitski and F. M. F. de Groot, *Micron*, 2010, **41**, 687.

17 T. Richter, K. Godehusen, M. Martins, T. Wolff and P. Zimmermann, *Phys. Rev. Lett.*, 2004, **93**, 23002.
